# *RCL1* copy number variants are associated with a range of neuropsychiatric phenotypes

**DOI:** 10.1038/s41380-021-01035-y

**Published:** 2021-02-17

**Authors:** Catherine A. Brownstein, Richard S. Smith, Lance H. Rodan, Mark P. Gorman, Margaret A. Hojlo, Emily A. Garvey, Jianqiao Li, Kristin Cabral, Joshua J. Bowen, Abhijit S. Rao, Casie A. Genetti, Devon Carroll, Emma A. Deaso, Pankaj B. Agrawal, Jill A. Rosenfeld, Weimin Bi, Jennifer Howe, Dimitri J. Stavropoulos, Adam W. Hansen, Hesham M. Hamoda, Ferne Pinard, Annmarie Caracansi, Christopher A. Walsh, Eugene J. D’Angelo, Alan H. Beggs, Mehdi Zarrei, Richard A. Gibbs, Stephen W. Scherer, David C. Glahn, Joseph Gonzalez-Heydrich

**Affiliations:** 1 Division of Genetics and Genomics, Boston Children’s Hospital, Boston, MA USA; 2 The Manton Center for Orphan Disease Research, Boston Children’s Hospital, Boston, MA USA; 3 Department of Pediatrics, Harvard Medical School, Boston, MA USA; 4 Tommy Fuss Center for Neuropsychiatric Disease Research, Boston Children’s Hospital, Boston, MA USA; 5 EPICenter, Boston Children’s Hospital, Boston, MA USA; 6 Department of Neurology, Boston Children’s Hospital, Boston, MA USA; 7 Department of Psychiatry and Behavioral Sciences, Boston Children’s Hospital, Boston, MA USA; 8 Division of Newborn Medicine, Boston Children’s Hospital, Boston, MA USA; 9 Department of Molecular & Human Genetics, Baylor College of Medicine, Houston, TX USA; 10 Baylor Genetics Laboratories, Houston, TX USA; 11 The Centre for Applied Genomics and Programs in Genetics and Genome Biology, The Hospital for Sick Children, Toronto, ON Canada; 12 Genome Diagnostics, Department of Paediatric Laboratory Medicine, The Hospital for Sick Children, Toronto, ON Canada; 13 Human Genome Sequencing Center, Baylor College of Medicine, Houston, TX USA; 14 Department of Psychiatry, Harvard Medical School, Boston, MA USA; 15 Department of Molecular Genetics and McLaughlin Centre, University of Toronto, Toronto, ON Canada; 16 Olin Neuropsychiatry Research Center, Institute of Living, Hartford Hospital, Hartford, CT USA

**Keywords:** Genetics, Psychiatric disorders

## Abstract

Mendelian and early-onset severe psychiatric phenotypes often involve genetic variants having a large effect, offering opportunities for genetic discoveries and early therapeutic interventions. Here, the index case is an 18-year-old boy, who at 14 years of age had a decline in cognitive functioning over the course of a year and subsequently presented with catatonia, auditory and visual hallucinations, paranoia, aggression, mood dysregulation, and disorganized thoughts. Exome sequencing revealed a stop-gain mutation in *RCL1* (NM_005772.4:c.370 C > T, p.Gln124Ter), encoding an RNA 3′-terminal phosphate cyclase-like protein that is highly conserved across eukaryotic species. Subsequent investigations across two academic medical centers identified eleven additional cases of *RCL1* copy number variations (CNVs) with varying neurodevelopmental or psychiatric phenotypes. These findings suggest that dosage variation of *RCL1* contributes to a range of neurological and clinical phenotypes.

## Introduction

Large-scale studies of common variants provide insight into the genetic architecture of psychiatric disorders such as schizophrenia and bipolar disorder [1, 2]. But even though these studies have involved hundreds of thousands of participants, they have typically explained only a small fraction of the genetic contribution to these focal illnesses, and have identified scores of loci that, with follow up, have pointed to putative risk genes [3–5]. Exome sequencing (ES) of unrelated cases with idiopathic schizophrenia (and controls) have identified gene sets involved in illness risk, but have not, with the possible exception of *SETD1A* and *UNC13B*, clearly identified specific risk genes [6–8]. And while common variant and ES studies with even larger samples will likely provide important insights into the genetic nature of psychiatric disorders, the advantages of other experimental designs should also be considered. For example, analyses of rare Mendelian forms of common diseases (e.g., extreme phenotypes) is an effective strategy to discover genes that influence idiopathic forms of illness [9, 10]. Among these, some may bear mutations that have a profound effect on the phenotype (‘large effect’ genes). Similarly, ‘genetics first’ approaches that focus on rare genetic disorders or recurrent copy number variants can provide candidate gene sets [9, 11, 12]. However, these approaches often provide several candidate genes within CNVs, and determining the most relevant gene for follow-up can be a daunting task [13, 14].

The increasing use of ES and genome sequencing (GS) in medical genetics clinics has spurred a dramatic increase in the number of individual cases reported in which highly penetrant genetic mutations are now associated with specific phenotypes [9, 14]. Collecting such data from sites around the world is now becoming more feasible and is enabling the systematic study of specific rare genotype-phenotype associations followed by subsequent biological experimentation.

However, there are a number of methodological issues that must be considered when analyzing rare genetic mutations in genes associated with severe Mendelian phenotypes.

Here, we utilize a clinical pipeline for identifying causal genes in Mendelian phenotypes, identifying a premature stop-gain in *RCL1* in a patient with very early onset psychosis (VEOP). While *RCL1* has not yet been reported in the context of psychotic symptoms, a rare missense variant in *RCL1* has been linked to an increased risk of depression [15]. Querying of two large academic medical centers for *RCL1* associated copy number variation (CNV) cases further identified 13 additional *RCL1* deletion and duplication patients with varying neurological and psychiatric phenotypes. We then performed human brain immunohistochemistry and transcriptome expression analyses across tissue types to demonstrate a cell type basis for *RCL1* expression in the brain. We conclude that dosage variation in *RCL1* may confer large effects on the brain and behavior.

## Methods (short methods)

**Fig. 1:**
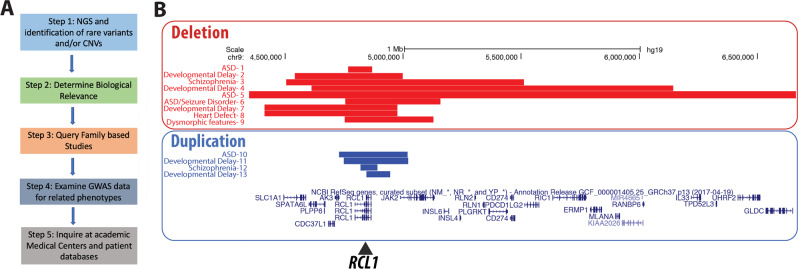
Framework and additional cases. **a** Study framework to verify candidate mutations resulting in severe Mendelian *RCL1* phenotypes. **b** *RCL1* copy number deletions (red) and duplications (blue) identified in academic medical centers in this study. Phenotypes consist of a range of neuropsychiatric features, individuals numbered 1–13. Note that individuals 8 and 9 were too young to display psychiatric symptoms. The genomic alignment panel includes genes surrounding *RCL1* on chromosome 9 (9p24.1), which consists of 11 exons encompassing 68 kb, and additional *RCL1* isoforms (color figure online).

We utilized a strategy for discovering genes of large effect in Mendelian forms of severe mental illness (see Fig. 1) that begins with next-generation sequencing and identification of rare variants or copy number changes across the genome, with an emphasis on known genes with neuropsychiatric involvement according to the published literature and internal databases, using well-defined filters and protocols [16, 17]. The biological relevance of the candidate variants in affected intervals was queried with help of the Allen Brain Atlas (https://portal.brain-map.org/) and other publicly available bioinformatics databases such as ClinGen (https://clinicalgenome.org/) and String (http://string-db.com/); model organism databases such as zfin (https://zfin.org/) were also used to obtain evidence for or against the variant/gene/interval’s hypothesized involvement in the phenotype. We also queried whether any family-based studies show segregation of genetic variants with phenotypes of interest and whether the gene or locus is involved in related phenotypes. GWAS data were examined to determine if the gene is a candidate for this phenotype or related phenotypes—because this process can be labor-intensive, we used aggregation tools such as https://www.ebi.ac.uk/gwas/home. For example, the overlap between psychiatric and neurodevelopmental phenotypes such as autism spectrum disorder (ASD), psychosis, and schizophrenia requires a broad literature search for a comprehensive investigation of whether the gene or locus might be involved in one of these neurological disorders. Finally, if there was evidence of possible involvement, we identified additional patients to support the relationship between gene/interval involvement by querying large academic medical centers that have databases of next-generation sequencing data and phenotypes or by using the Matchmaker exchange platform (https://www.matchmakerexchange.org/) [18].

### Human subjects and samples

Research performed at Boston Children’s Hospital on human samples was conducted according to protocols approved by the institutional review board of Boston Children’s Hospital. Subjects were evaluated and identified in the Boston Children’s Hospital Developmental Neuropsychiatry clinic, and written as well as verbal consent/assent was obtained under the Gene Discovery Core protocol of the Manton Center for Orphan Disease Research. Patient data has been deposited into DECIPHER (https://decipher.sanger.ac.uk/patient/421796/). De-identified querying of samples at two other large academic medical centers (Baylor College of Medicine/Baylor Genetics and The Hospital for Sick Children, Toronto) was performed as per institutionally approved methods.

### Proband family next-generation sequencing and chromosomal microarray analysis (CMA)

The patient underwent phlebotomy for CMA of peripheral blood lymphocytes. Genomic DNA was examined by array-based comparative genomic hybridization (aCGH) using the ClariView Array (Claritas Genomics, Cambridge, MA). The array contains DNA oligonucleotide probes in or flanking most exons of the evaluated genes and is designed to detect most single-exon deletions and duplications. Probe sequences and locations are based on Genome Reference Consortium build 37 (GRCh37)/UCSC hg19. Data analysis was performed with Agilent CytoGenomics 5.0.0.38 software (Cambridge, MA).

The proband and both parents underwent ES at the Yale Center for Mendelian Genomics. Whole-exome libraries were prepared using the KAPA Hyper kit, and the products were enriched with IDT’s xGen Exome Research Panel v1.0 using multiplexed capture of 16 samples. Exomes were sequenced on an Illumina HiSeq 4000 using paired-end chemistry at a read length of 100 bp. FASTQs were aligned by Codified Genomics (proprietary algorithm, Houston, TX). *De novo*, homozygous, heterozygous, and rare variants were examined if they passed quality criteria adapted from Yuen et al. [16]: (1) read depth in the proband and parents ≥10X; (2) allele frequencies <1% in all population databases; (3) residual variation intolerance score (RVIS) percentile <70 and loss-of-function observed/expected upper bound fraction (LOEUF) <0.6 [19]; and (4) read ratio ≥25% of the alternate allele in the proband of the trio.

### Human tissue brain preparation and immunohistochemistry

Control fetal (21 weeks gestation), flash froze neonatal (270 days post-birth), and adult cerebral cortex samples were prepared as previously described in Smith et al. [20]. Briefly, samples were sectioned at 20–30 μm thickness (Leica Cryostat) and mounted immediately onto warm charged SuperFrost Plus slides (Fisher). Whole tissue imaging was performed on a Zeiss Axio Observer or LSM710 confocal with image tiling at 20X magnification. Images were uniformly corrected by Zen Blue Software during stitching together. Following fixation (4% PFA), primary antibodies included mouse anti-RBFOX3 (NeuN, Millipore) and rabbit anti-RCL1 (Sigma), and secondary antibodies included AlexaFluor (1:500; 488 nm, 563 nm) donkey anti-mouse and donkey anti-rabbit (Invitrogen). See Supplemental Table 1 for details.

**Table 1:** Variants of interest in the index patient.

Gene	Genomic location (hg19)	Variant type	HBVS protein	Allele count in gnomAD	Zygosity	Parent of origin	Variant interpretation (ACMG/AMP guidelines via InterVar) [43]
*CAPN1*	11:64975716 A > G, ENST00000527323.1:c.1712A > G	Cryptic splice (Donor)	p.Asn571Ser	4/ 210744	Het	Pat	VUS
*CAPN1*	11:64977855 C > T, ENST00000527323.1:c.1991C > T	Nonsynonymous	p.Ser664Leu	3/ 247776	Het	Mat	VUS
*RCL1*	9:4827019 C > T, ENST00000381750.4:c.370 C > T	Stop-gain	p.Gln124Ter	0	Het	Pat	Pathogenic

### Human RNA expression analysis

The Allen Human Brain Atlas maintains an online database of transcriptional expression profiling across cortical brain regions from age 8 weeks post-conception to adult [21]. BrainSpan data analysis of *(ENST00000381750.9)* at *chr9:4792944-4861066* was referenced on November 2, 2019. RNA-seq expression measured in RPKM (reads per kilobase exon per million mapped reads) was obtained from the BrainSpan project data and summarized to Gencode v10 exons for all annotated neocortex tissues aged 8 weeks post-conception to 38 years. See Supplemental Table 1 for details.

## Results

### Clinical findings in the proband and affected father

The proband is currently an 18-year-old man who at 14 years of age experienced neuropsychiatric symptoms over the course of a year that accelerated 4 months prior to his presentation with catatonia, auditory and visual hallucinations, paranoia, aggression, mood dysregulation, and disorganized thoughts. An extensive medical evaluation was performed due to the accelerated nature of the patient’s decline just prior to presentation with catatonia. The patient had a relatively unremarkable development before his psychiatric hospitalization at age 14. He was born full-term after an uncomplicated pregnancy. He had some articulation problems that were corrected with speech therapy. He required tympanostomy tubes as an infant. All other developmental milestones were achieved on time. The patient began experiencing an increase in anxiety starting at age 13. At age 13 years and 9 months, he had a syncopal episode that was evaluated with an electroencephalogram (EEG) which showed intermittent left temporal slowing. However, brain magentic resonance imaging (MRI) obtained at this time was normal. After two additional near syncopal episodes, he was evaluated by a neurologist who concluded that the episodes were not likely seizures. He had previously experienced a head injury without loss of consciousness while playing sports. Prior to his decline in functioning, he was an above-average student and varsity athlete with no history of a learning disability, attention-deficit/hyperactive disorder (ADHD), or special education services.

As the patient’s anxiety increased during the year leading up to his first clearly psychotic episode, his grades declined, and he lost interest in activities that he had once enjoyed. Shortly thereafter, the patient presented with psychosis and catatonia and was hospitalized at age 14. Due to catatonia, an evaluation for autoimmune and other neurologic causes of psychosis was performed. His cerebrospinal fluid (CSF) was found to have elevated protein of 80 mg/dl (normal range: 15–45 mg/dl), which was consistently elevated on four serial repeat lumbar punctures. An F-18-fluoro-2-deoxyglucose positron emission tomography scan showed mild to moderate midbrain and frontal hypometabolism that was regarded as abnormal but nonspecific. The rest of these evaluations, including repeat EEG and MRI as well as serum and CSF autoantibody screens and metabolic studies, were normal.

The elevated CSF protein prompted concern for an autoimmune cause for the patient’s condition. He was treated with methylprednisone and intravenous immunoglobulin but proceeded to symptomatically worsen. Before immune or antipsychotic therapy, he responded to benzodiazepines with some improvement in global function from approximately 20% of baseline to 65% of baseline. However, the patient continued responding to internal stimuli and hallucinations. He had an inadequate response to several antipsychotics and achieved his best response to combined treatment with clozapine and paliperidone. While the patient’s hallucinations and delusions are controlled on this treatment, his thoughts have remained somewhat disorganized. He has had difficulty with urinary frequency and incontinence possibly attributable to a side effect of clozapine. Repeat EEG at age 17 showed mild generalized background slowing and absence of a posterior dominant rhythm consistent with a mild encephalopathy, possibly attributable to his underlying condition and/or effect of clozapine.

Neuropsychological evaluations were completed 4, 12, 29, and 44 months after his functional regression began at age 14 years with his latest evaluation completed at age 17 years 10 months. These evaluations found that intellectual functioning was in the low average range and remained consistent between testing episodes. His full-scale IQ across the four evaluations was 82/87/83/78, respectively, with the differences not considered significant. Comparing the initial evaluations at 4 and 12 months to the final evaluation, performance on formal measures of attention, graphomotor output speed, auditory verbal learning, and memory improved. Word retrieval and graphomotor output speed were within age expectations at the last evaluation. Scores on a measure of working memory were stable. Comparing the final two evaluations, he had much more difficulty focusing, sustaining attention, and encoding/learning at the final evaluation. However, at the final evaluation, he was able to hold on to the information he learned and had better scores on recognition items. His neuropsychologist concluded that his scores on measures of verbal learning/memory did not support decay in memory, but rather an impairment in encoding/learning likely secondary to attention and executive function deficits as well as a slow rate of information processing.

His catatonia has remitted with clonazepam. He meets DSM-5 criteria for schizophrenia, with a history of unspecified depressive disorder and catatonia as confirmed by the structured clinical interview for DSM-5 research version (SCID-5-RV) [22]. Repeated neuropsychological examinations undertaken after his functional decline found low average intelligence with deficits in sustained attention, processing speed, fine motor speed, dexterity in both hands, verbal memory encoding, and verbal fluency.

The patient’s family history is notable on his father’s side for early cognitive decline, multiple sclerosis (MS), anxiety, depression, alcoholism, obsessive-compulsive disorder, bipolar disorder, and suicidal ideation. His father is reported to have emotional regulation and anger control difficulties that worsened through mid-adulthood though he has not been clinically diagnosed. The patient’s paternal grandfather has had an early cognitive decline that was initially noted after a stroke at age 61 years and has continued to progress. His paternal aunt has had multiple psychiatric hospitalizations, and another paternal aunt has MS. On his mother’s side, there is a psychiatric family history of anxiety and dementia in the maternal great grandmother (the patient’s mother’s paternal grandmother) that started in her 70 s and also affected her siblings in their 70 s.

### Identification of *RCL1* stop-gain paternally inherited variant and additional variants

ES showed heterozygosity for a paternally inherited early stop codon in the *RCL1* gene ([OMIM # 611405] NC_000009.11:g.4827019 C > T, NM_005772.4:.370 C > T, NP_005763.3:p.Gln124Ter). This variant has not been recorded in any database. However, a private Gln124His variant was identified in an internal cohort of patients with interstitial cystitis. No structural variants involving *RCL1* were identified in the human gene mutation database and no gold-standard variants involving *RCL1* were listed in the database of genomic variants (DGV) (September 18, 2020).

The patient was also compound heterozygous for two variants in the *CAPN1* gene: NC_000011.9:g.64977855 C > T, NM_001198868.1:c.1991C > T, NP_001185797.1:p.Ser664Leu (Sift = 0.0, Polyphen2 = P, MutationTaster = disease-causing), and NC_000011.9:g.64975716 A > G, NM_001198868.1:c.1712A > G, NP_001185797.1:p.Asn571Ser (Sift = 0.04, Polyphen2 = D, MutationTaster = disease-causing). All variants were confirmed by Sanger sequencing in the trio (see Table 1).

The patient’s compound heterozygous variants in *CAPN1* were classified as variants of uncertain significance (Table 1). *CAPN1* (OMIM # 114220) encodes calpain-1, a large subunit of μ-calpain, a calcium-activated cysteine protease widely present in the central nervous system. Mutations in *CAPN1* have been linked to hereditary spastic paraplegia type 76, which is characterized by adult-onset, chronically progressive corticospinal tract dysfunction (SPG76) with variable cerebellar dysfunction, peripheral neuropathy, and urinary symptoms including incontinence [23–25]. At the most recent follow-up, the patient has not manifested any signs of pyramidal tract dysfunction (spasticity, hyperreflexia, abnormal plantar response), cerebellar dysfunction/ataxia, or peripheral neuropathy, although we cannot exclude that these may later develop with age. Thus, we are not able to rule out that the compound heterozygosity of *CAPN1* mutations is contributing in some way to the patient’s presentation (e.g., urinary symptoms).

The patient’s clinical microarray (CMA) revealed a paternally inherited copy number loss in 2q13 (Min/Max coordinates [hg19]: 110862474-110983457; 110833650-111406694; Min/Max Size: 120 kb/573 kb, containing genes *MALL*, *NPHP1*, *LIMS3*, *RGPD6*, and *BUB1*). Mutations of *NPHP1* (OMIM #607100) are associated with autosomal recessive nephronophthisis. A sequence variant in the other allele was not seen on ES. There are also overlapping deletions listed in the DECIPHER database (e.g., cases 337636, 271516, 258446, 259866, 259955, 308018, and 283832). Associated features include ASD, intellectual disability, cognitive delay, global developmental delay, delayed speech, central hypoventilation, and dysphagia. A copy number loss encompassing a similar interval (including *MALL*, *NPHP1*, *LIMS3*, *RGPD6*, and *BUB1*) has been reported in ClinVar as a variant of uncertain significance with the phenotype of developmental delay and facial dysmorphisms (SCV000709783.2). A similar interval to the one reported in our proband with the exception of *BUB1* was present in DGV gold-standard variants (defined as curated variants from a selected number of studies in DGV) with a frequency of 0.07% (gssvL65158, phenotype unknown). 2q13 deletions encompassing *NPHP1* are present in 0.571% of controls in the UK Biobank (865 in 151,619 individuals) and 0.571% of other controls (152 in 26,628 individuals) [26].

The premature stop codon in the *RCL1* index case and the 2q13 copy number loss are paternally inherited. There is a paternal family history of psychiatric hospitalizations and difficulties with emotional regulation in the father and grandfather.

### Blood phenotypes

Common variants in *RCL1* affect red blood cell and platelet indices, including significantly decreasing or increasing mean corpuscular volume (MCV), mean corpuscular hemoglobin (MCH) [27], and red blood cell count (RBC), affecting platelet count (decreasing [28, 29] and increasing [30, 31]) and increasing platelet crit [30] (defined as platelet count × mean platelet volume [MPV]/10,000). Therefore, blood panel values were abstracted from the proband’s medical record, including 20 assessments over the course of over 3 years. While MCV was in the normal range, MCH was low at 27.2 ± 0.37 picograms/cell (normal range 28.2–30.5), and his RBC was elevated at 5.2 ± 0.22 million cells per µL (normal range 3.7–4.9). In addition, MPV was elevated with a mean of 11.3 ± 0.55 fL (normal range 7.5–8.3). In agreement with previous *RCL1* blood phenotypes, the proband’s *RCL1* variant is likely contributing to the shifted MCV, RBC, and MPV phenotypes.

### Robust *RCL1* transcriptional expression during human development

**Fig. 2:**
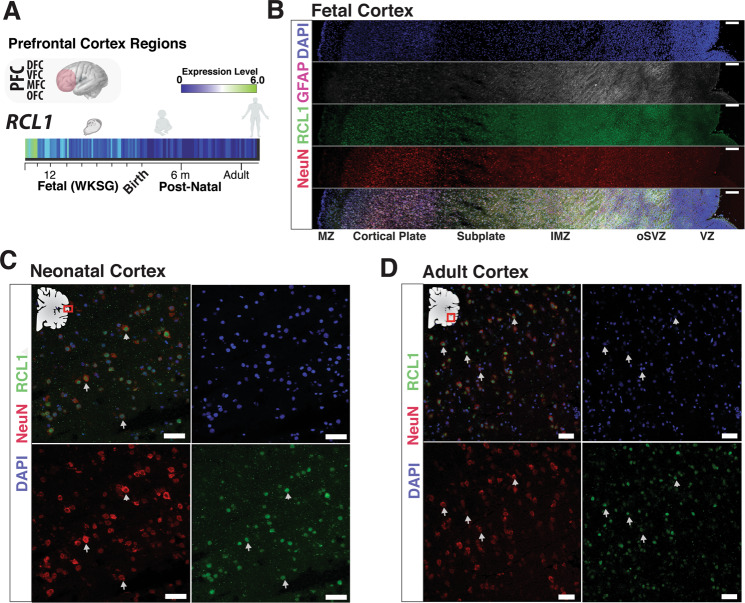
*RCL1* expression in human brain development. **a** Bulk transcriptome analysis of prefrontal cortical regions demonstrates *RCL1* transcripts enriched during gestational weeks (WKSG) and decrease postnatally, presented as log_2_ RPKM (reads per kilobase per million) values. DFC dorsolateral prefrontal cortex, VFC ventrolateral prefrontal cortex, MFC medial frontal cortex, OFC orbital frontal cortex. Transcriptome data from Allen Institute for Brain Science Atlas. **b** Confocal fluorescence image of a mid-gestation human coronal fetal cortex tissue with RCL1 antibody co-labeling with markers, glial fibrillary acidic protein (GFAP), a neuronal marker (NeuN), and nuclei marker (DAPI). RCL1 is lowly, yet uniformly present across developing cortex layers, with an abundant signal within the cortical plate. Scale bar 100 µm. MZ marginal zone, IMZ intermediate (fiber) zone, oSVZ outer sub-ventricular zone, VZ ventricular zone. RCL1 and cell type specific antibody staining with corresponding confocal fluorescence imaging of (**c**) 9-month-old human cortex (neonatal) and (**d**) adult cortex (37-year-old). Neuronal marker (NeuN) and global nuclei marker (DAPI) show RCL1 present in both neurons and non-neuronal cell types. Arrows indicate cells colocalized for NeuN and RCL1. Scale bar, 50 µm.

*RCL1* demonstrates robust RNA expression in the prefrontal cortical regions during early human development which extends into the neonatal period and subsequently decreases into adulthood (Fig. 2a). To evaluate RCL1 localization in the developing human cortex, we performed immunohistochemical analyses of RCL1 in the mid-gestation fetal cortex (20 weeks gestation) which demonstrate that RCL1 is ubiquitious across the cortical column (ventrical zone to cortical plate), with robust expression in deep-layer CTP2 + neurons (cortical layer 5/6) and among cells in the ventricular zone, including GFAP + progenitors (Fig. S1). However, RCL1 did not colocalize with GFAP + fibers located in more superficial cortical regions (Fig. S2c). Immunohistochemical analyses of the human neonate cortex (270 days post-birth) and adult cortex (37 year old) revealed the highest RCL1 signal colocalized with a neuronal marker (NeuN, Fig. 1) with the limited signal in the white matter (Fig. S1a). In agreement with IHC data, analysis of adult human neocortex single cell RNA sequencing data demonstrates that *RCL1* is broadly expressed across several neuronal types, including enrichment within specific excitatory and inhibitory neuron clades (Fig. S2).

### Identification of additional patients with copy number variants including *RCL1*

To determine if the observed *RCL1* variant could cause the proband’s presentation, we queried cases undergoing clinical CMA testing at two major medical centers for individuals with CNVs impacting *RCL1*. The Hospital for Sick Children’s population (*N* = 44,113 total individuals) is from various published and unpublished sets including MSSNG, the database of clinical variants, and microarrays. The Baylor genetics cohort consists of ~58,000 total individuals, containing probands of diverse phenotypes and parental samples. Thirteen patients were identified with CNVs encompassing *RCL1*; nine with copy number losses and four with copy number gains (Tables 2 and 3 and Fig. 1).

**Table 2:** Additional *RCL1* copy number losses: cases from academic medical centers.

Case #	Inheritance	Start (hg19)	End (hg19)	Size	Copy number change	Age	Phenotype
Toronto SickKids*							
1	Paternal	4,767,677	4,869,801	102 kb	Loss	Not available	Autism spectrum disorder (ASD), inherited from father
2	Unknown, seen in sibling (unknown phenotype)	4,542,988	4,999,498	457 kb	Loss	Not available	Developmental delay, 2q13 duplication
3	Unknown	4,504,448	5,510,644	1006 kb	Loss	Not available	Schizophrenia
4	Unknown	203,861	6,648,114	6,444,253	Loss	Not available	Developmental delay
5	Unknown	4,611,869	6,144,065	1,532,196	Loss	Not available	Developmental delay, ASD
**Baylor genetics**							
6	Paternal	4,744,770	5,037,925	293 kb	Loss	19 years	ASD, seizure disorder
7	Paternal	4,428,574	4,979,623	551 kb	Loss	6 years	Moderate developmental delay, intellectual disability, dysmorphic features
8	Unknown	4,428,574	4,979,623	551 kb	Loss	0 months	Congenital heart defect, small deletion of *PABPC4L*
9	Unknown	4,781,753	5,015,889	234 kb	Loss	4 months	Dysmorphic features, cleft lip and palate

*SickKids samples were either unpublished clinical samples or those from their publications [69, 70].

**Table 3:** Copy number gains in *RCL1* from Academic Medical Center 1 (Toronto SickKids*).

Case #	Inheritance	Start (hg19)	End (hg19)	Size	Copy number change	Age	Phenotype
10	Paternal	4,725,823	5,012,037	286 kb	Gain (×3)	Not available	ASD
11	Unknown	4,744,779	5,015,759	271 kb	Gain (×3)	Not available	Developmental delay and ADHD; pathogenic 16p11.2 copy number loss
12	Unknown	4,814,948	4,886,266	71 kb	Gain (×3)	Not available	Schizophrenia
13	Unknown	4,841,229	4,938,706	97 kb	Gain (×3)	Not available	Developmental delay

*SickKids samples were either unpublished clinical samples or those from their publications [69, 70].

## Discussion

We present an index case of an 18-year-old male whose decline in functioning starting at age 14 led to a DSM-5 diagnosis of schizophrenia with a history of catatonia and unspecified depressive disorder. ES revealed a paternally inherited premature stop codon in *RCL1* p.(Gln124Ter), a gene with CNVs identified in 13 additional individuals, 11 of whom have neuropsychiatric phenotypes.

### Biological evidence and analysis of *RCL1* in human tissue

*RCL1* is a ubiquitously expressed, highly conserved eukaryotic gene located on chromosome 9 (9p24.1) involved in ribosome biogenesis [32–37]. *RCL1* is expressed globally across tissues (ENSG00000120158.11; https://gtexportal.org/home/gene/RCL1), with the highest expression levels in the liver, adipose, and arterial tissues. Defects in ribosomal biogenesis have been reported to result in a range of phenotypes, including embryonic lethality, growth delays, craniofacial defects, sterility, skin and skeletal abnormalities, anemia, cirrhosis, and cognitive impairment [38, 39]. A review of data in gnomAD (v2.1.1, accessed 04/03/2020) identified only six presumptive loss-of-function mutations in *RCL1* (20.6 expected), resulting in a LOEUF of 0.57 [19]. The presence of only six individuals heterozygous for *RCL1* loss-of-function mutations among the 141,456 supposedly healthy individuals in the gnomAD cohort is consistent with the pathogenicity of *RCL1* haploinsufficiency under a model accounting for later onset and variable expressivity of a psychiatric condition.

Archived Zfin data of the *RCL1* zebrafish orthologue *rcl1* (transgenic insertion rcl1 hi2452Tg zebrafish, ZFIN ID: ZDB-GENE-040930-11) lists phenotype “day 2: slightly smaller head and eye; day 5: small head and eyes, underdeveloped liver/gut, a little pericardial edema” [40]. While no behavioral phenotype or seizure activity is listed, it is interesting that smaller head size is a documented phenotype of zebrafish *rcl1* disruption [41], and *rcl1* was one of 315 genes identified in the screen as being essential for zebrafish development (Dr. Adam Amsterdam, personal communication). Similar to human data presented here, the zebrafish *rcl1* gene is highly expressed *in utero* (See Fig. 2a) consistent with the hypothesis that a significant early neurodevelopmental component carries the risk for schizophrenia spectrum disorders, deriving from processes operating in prenatal development [42].

### RCL1 expression is enriched to neurons in the neonatal and adult human brain

Our postnatal human neocortex IHC experiments indicate that *RCL1* is primarily expressed in nuclei of neurons, supporting its role as a RNA 3′-terminal phosphate cyclase-like protein involved in ribosome biogenesis, with some expression in the cytoplasm as well. However, we did not observe *RCL1* colocalized with layer 1 interlaminar astrocyte processes as previously reported in the adult cortex [15], nor did we find significant expression within the adult white matter layer or within GFAP + fibers. Taken together, our RNA expression analysis and IHC data demonstrate that *RCL1* is likely enriched in neurons during the neonatal and adolescence period, offering a pathological basis for early life psychosis. Further exploration of *RCL1* expression in the adult cortex at the single-cell transcriptome level shows *RCL1* expression in both excitatory and inhibitory neuronal subtypes, without expression in non-neuronal cells, suggesting that *RCL1* dysfunction could disrupt cell types differentially.

### Family-based studies

A recent GS study focused on the identification of rare variants in families from an isolated population reported a missense p.Leu372Phe variant (rs115482041) in *RCL1* segregating with depression in a multi-generation pedigree [15]. The variant showed significant association with depressive symptoms (*N* = 2393, *β*_T-allele_ = 2.33, *P-*value = 1 × 10^−4^) and explained 2.9% of the estimated genetic variance of depressive symptoms (22%) in the genetically isolated population. Both of the homozygous carriers exhibited high scores on the depressive symptom rating scale and were also diagnosed with major depressive disorder requiring a combination of psychotherapy and antidepressant treatment. Despite the variant being twice as rare in a neighboring outbred population (MAF < 0.5%), the same variant showed a similar effect and significant association with depressive symptoms (*N* = 1604, *β*_T-allele_ = 3.60, *P*-value = 3 × 10^*−*2^) [15].

This missense change in Rs115482041of Leu to Phe is predicted to be Likely Benign using ACMG criteria [43]. This is in contrast to the variant discussed in our proband, which results in a premature truncation of the protein and is thus scored as Pathogenic using ACMG criteria. In accordance with Mort et al., which predicted that genes important in binding have an abundance of missense changes [44] (*n* = 214.7 expected in gnomAD for RCL1, and 258 observed) and a paucity of nonsense changes (20.6 expected, 6 observed), we conclude that *RCL1* binds partners during ribosomal biogenesis [45]. The evidence that the amino acid change in Rs115482041 confers a risk of a psychiatric phenotype supports the hypothesis that a nonsense mutation in *RCL1* could also increase risk of neuropsychiatric phenotypes.

### Investigation of GWAS studies

*RCL1* variants have been reported to influence red blood cell volume [27, 46], platelet count [31, 47], and platelet crit [30] in several genome-wide association studies, suggesting a role for *RCL1* in disorders of the blood. Our patient shows an elevation of MPV, providing supporting evidence that the RCL1 variant is physiologically active and producing a known associated phenotype.

RCL1 may be involved in the response to antipsychotic treatment in schizophrenia [48], and a case-control study has shown that an intergenic SNP variant between the *HNRNPA1P41* pseudogene and *RCL1* is statistically significantly associated with Alzheimer’s disease (AD) and related dementias (rs9969783 chr9:4,888,441 [hg19], effect size: −0.2949 [unit decrease] std dev: 0.0615 *p*-value: 1.63E−06) [49]. Psychosis occurs in up to 50% of individuals with AD and is associated with significantly worse clinical outcomes [50, 51]. Atypical antipsychotics are sometimes used in AD, suggesting shared mechanisms [52], and similar neuropsychological deficits in processing speed and executive function have been observed in individuals with very late onset schizophrenia-like psychosis and AD + psychosis [53]. In addition, a recent study found that a schizophrenia polygenic risk score was associated with AD + psychosis [54]. These new findings point towards psychosis in AD sharing some genetic liability with schizophrenia and are consistent with the hypothesis that dysregulation of *RCL1* is related to various brain-related illness phenotypes.

### Additional patients with copy number variants including *RCL1*

Through collaborative data sharing with major academic medical centers, we were able to identify additional individuals with CNVs in our candidate gene, many of whom had neuropsychiatric phenotypes. Two small copy number losses have childhood-onset psychosis and ASD as their phenotypes. Two of the youngest patients with copy number losses in *RCL1* did not have diagnoses of developmental delay or psychiatric phenotypes, though it was too early in their lives for those phenotypes to be recorded.

Nine additional unrelated probands who harbor CNVs involving the *RCL1* gene were identified at Toronto SickKids (Tables 2 and 3). Seven probands in this group have been diagnosed with significant neurodevelopmental phenotypes and two with schizophrenia. The two probands diagnosed with schizophrenia have a duplication and deletion in the *RCL1* gene, respectively. The copy number gain with the smallest interval (71,319 bp and duplicating exons 2–9, Patient #12) has a schizophrenia phenotype. However, this CNV’s impact on function is unknown, as it is overlapping one end of the gene and leaving one copy intact. One other patient with a developmental delay has a duplication interval overlapping one end of the gene spanning exons 6–9 (Patient #13), which may also leave a copy of the gene intact. Unfortunately, recontacting patients for a more detailed history or additional samples was not possible.

At Baylor genetics, four patients were identified with copy number loss of *RCL1* smaller than one megabase. One patient (Patient #8, originally diagnosed with a cardiac defect and a carrier of an additional *PABPC4L* deletion*)*, is now 10 years of age with no reported neuropsychiatric phenotype. Another patient (Patient #9) was too young at the time of testing to have shown a psychiatric phenotype (at 4 months) and an update was not available. The other two patients have ASD and seizures or moderate developmental delay, intellectual disability, and dysmorphic features, respectively.

Several of the academic medical center cases are also paternally inherited, and some harbor additional CNVs. The first (Patient #11) has a CNV in 16p11.2, which is known to have widely varying phenotypes, ranging from schizophrenia to developmental delay and intellectual disability to no remarkable phenotypes at all [55]. Another (Patient #2) has a reciprocal 2q13 duplication. While the index case has a 2q13 copy number loss, this patient has developmental delay and a 456,511 bp CNV loss encompassing *RCL1* and an additional 2q13 duplication. It is possible that a second genetic mutation or CNV is required to unmask the predisposition to a neuropsychiatric phenotype, in accordance with Girirajan et al. [56].

ClinGen does not list any benign gains or losses encompassing *RCL1* but lists several pathogenic gains and losses, with phenotypes including developmental delay, low-set ears, abnormal gait, facial abnormalities, ASD, and intellectual disability.

### Loss of *MALL, NPHP1, LIMS3*, and *BUB1*

Chromosome 2q13 deletions and duplications are associated with developmental delay as well as psychiatric and behavioral disorders [57, 58]. The 2q13 copy number loss is associated with ASD, but in this patient, only the *BUB1* gene overlaps in an autism-associated 2q13 interval described by Guivarch et al. [59]. Mutations in *BUB1* can cause mosaic variegated aneuploidy and increase the risk of colorectal cancer at a young age [60]. A paper describing individuals with various overlapping intervals involving *RGPD6* and *BUB1* reported one individual as having normal neuropsychiatric development but described others with developmental delay/intellectual disability, ASD, and  pervasive developmental disorder - not otherwise specificed (PDD-NOS) [61]. Moreover, given the above-average athletic and scholastic abilities of the proband before his first psychiatric episode, and the CNV’s frequency among control sequencing databases (0.571% in the UK Biobank), it is difficult to conceive that the 2q13 interval is causative to the patient’s psychosis phenotype. Lastly, the patient’s interval also does not include candidate genes for schizophrenia in the 2q13 region (*ANAPC1*, *BCL2L11*, or *MERTK)* mentioned in Costain et al. [62].

### CAPN1

The proband also has compound heterozygous missense mutations in *CAPN1*, which may be resulting in an atypical version of the Mendelian disease spastic paraplegia-76 (SPG76), in particular since this subtype is associated with young adult spastic paraplegia onset. While SPG76 is not traditionally associated with early-onset psychosis, an SPG76 case report details psychosis in one patient at age 45, 20 years after the onset of lower limb stiffness and gait instability. This patient had a more deleterious homozygous nonsense *CAPN1* mutation (p.Trp392*), and was wheelchair-bound at age 40 years [63]. The individual had chronic depression (with a positive family history), and at age 45 years manifested severe cognitive and behavioral disturbances mimicking the clinical and neuroimaging features of behavioral variants of Frontotemporal Dementia, which include moderate cortical atrophy [63]. She also experienced urinary incontinence and urgency, though the onset of the urinary symptoms was 13 years after the onset of gait instability and lower limb stiffness. Currently, there are over 80 genetic subtypes of SP resulting in a spectrum of associated phenotypes, including the *SPAST* gene in SPG4, a microtubule-associated gene, which is associated with an increased rate of schizophrenia and psychosis in the affected individuals [64].

For the proband in this study, the paternal *CAPN1* variant (p.Asn571Ser) is extremely rare with an allele count of 4 in gnomAD (*n* = 210,744), with 2/4 of these *CAPN1* carriers belonging to the gnomAD psychiatric cohort. Similarly, the maternal variant (p.Ser664Leu) is present in three individuals in gnomAD (*n* = 247776), one of whom belongs to the psychiatric cohort. While CAPN1 is relatively tolerant to missense changes (gnomAD, o/e ratio of 0.78), both of the proband’s missense *CAPN1* variants are in the top 1% of deleteriousness as both have CADD scores above 24. *CAPN1* is a recessive gene and previous pathogenic variants have both been homozygous and compound heterozygous variants. ClinVar lists 35 reports of *CAPN1* mutations: 11/35 are pathogenic, 20/35 are (likely) benign and 4 are either VUS or have conflicting interpretations of pathogenicity. In terms of mutation type distribution in ClinVar, 8/11 of the pathogenic variants are loss of functions, two are missense and one is a non-essential splice site. Thus, the evidence for the compound heterozygous missense mutations in *CAPN1* to cause SPG76 in this patient is strong but not as compelling as if one of the variants were a loss-of-function. However, we cannot rule out that the proband is pre-symptomatic or oligosymptomatic for SPG76, although he does not yet have the core feature of leg spasticity. It is possible these phenotypes will develop over time; reports of SPG76 in the literature state age of onset ranging from 15 to 45 years [24, 63, 65–68]. Electrophysiology tests such as EMG/NCS or lower limb SSEP’s have not yet been performed on this patient, given the normal motor exams in the past and the likely difficulty of tolerating such tests with his psychiatric phenotype.

### Limitations

Our inability to recontact the additional cases prevented us from comprehensively phenotyping the patients, and we were unable to follow up on cardiac abnormalities and dysmorphic features that were noted in some patients and may have been present but unreported in others. Ideally, the patients should have been monitored longitudinally. In addition, consenting limitations prevented the querying of additional potentially causative mutations in CNV carriers.

## Conclusions

VEOP is an extreme phenotype of the more common adult-onset psychosis, thus making VEOP a candidate for Mendelian inheritance patterns. Mendelian disorders are more likely to involve genes of large effect. Therefore, we began with an index case of VEOP and utilized our systematic approach to identifying candidate genes for further investigation. Having established the involvement of *RCL1* in the brain and behavior of our index VEOP patient, we queried two academic medical centers and confirmed the involvement of *RCL1* in 13 additional patients with neurological disorders. *RCL1* deletions and duplications were associated with a range of neurological and psychiatric phenotypes. Copy number loss of *RCL1* was associated with developmental delay, intellectual disability, ASD, seizures, and schizophrenia in two separate patient populations at the two medical centers. Copy number gain of *RCL1* was associated with developmental delay, ASD, schizophrenia, and ADHD.

Our multistep study design had several advantages. Next-generation sequencing of rare Mendelian forms of complex disease increases the chances of discovering genes of large effect. Examination of model organism data on *RCL1* supports the hypothesis that disruption of *RCL1* results in a neurological phenotype. In addition, we used the Allen Brain Atlas database to screen RNA expression and validated these results through in-house functional studies to gain biological insights. Our screening included cell type RCL1 expression patterns from neonatal life to adulthood and offers a pathological transcriptional timeline that aligns with early developmental processes leading up to the psychosis period.

In conclusion, our findings identify *RCL1* as an important candidate gene for a range of neurological and psychiatric phenotypes, and we highlight published evidence that RCL1 protein is, in fact, present in the developing human brain. Our findings will undoubtedly guide future investigations into the genetic and pathological bases of neuropsychiatric phenotypes observed in the *RCL1* cases. Details of the etiology, neurobiological manifestations, and the role of other genetic loci or nongenetic effects in shaping phenotypes are yet to be elucidated.

## Supplementary information

Supplemental Information

## Data Availability

Schizophrenia sample IDs (from Psychiatric Genomic Consortium): Ed_PT-267W@cas_scz_edin_eur_A6.0*Ed_PT-267W. PT-BJMD@fam_scz_butr_eur_A6.0*1403_0_pca. Proband patient data has been deposited into DECPHER https://decipher.sanger.ac.uk/patient/421796.
