# Supplementary material for: RCL1 copy number variants are associated with a range of neuropsychiatric phenotypes
Source: Mol Psychiatry. 2021 Feb 17;26(5):1706–18. doi: 10.1038/s41380-021-01035-y (PMC8159744; doi:10.1038/s41380-021-01035-y)
Supplement: Supplementary file 1 — Supplemental Information [file 41380_2021_1035_MOESM1_ESM.docx]

**CONTACT FOR REAGENT AND RESOURCE SHARING**

Further information and requests for resources and reagents should be directed to and will be fulfilled by Lead Contact Catherine Brownstein (catherine.brownstein@childrens.harvard.edu).

**EXPERIMENTAL DETAILS**

**Human subjects and samples**

Individuals presented herein were identified and evaluated in a clinical setting, and biological samples collected after obtaining written informed clinical and/or research consent. Human subject research was conducted according to protocols approved by the institutional review boards (IRB) of Boston Children’s Hospital and Beth Israel Deaconess Medical Center. Fetal brain tissue was received after release from clinical pathology, with a maximum post-mortem interval of 4 h. Cases with known anomalies were excluded. Tissue was transported in Hibernate-E medium (Thermo Fisher) on ice to the laboratory for downstream processing. The neonatal and adult brain samples were obtained from the University of Maryland Brain and Tissue Bank of the NIH NeuroBioBank (sample number UMBN 5817) and stored at -80C until further processing.

**Human Tissue Brain Preparation and Immunohistochemistry**

Frozen cerebral cortex tissue were sectioned at 20 – 30 μm thickness (Leica Cryostat) and mounted immediately onto warm charged SuperFrost Plus slides (Fisher), then dried at -20°C for 20 minutes before storage at –80°C. After applying a hydrophobic barrier around the tissue (ImmEdge Pen, Vector Labs). Whole tissue confocal fluorescent imaging was performed on a Zeiss Axio Observer with image tiling at 20X magnification, and stitched together using Zen Blue Software. Standard image processing was completed using ImageJ and Zen sotware tools, including uniform intensity adjustments, and noise filtering (uniform despeckling and outlier removal).

**Supplemental Table 1. Sources**

| **Biological Samples** |  |
| --- | --- |
| Human Adult Cortex Tissue, control | UMD BioBank |
| Human Neonatal Cortex Tissue, control | UMD BioBank |
| Human Fetal Tissue | This Study |
|  |  |
| **Data** |  |
| Bulk Cortical Transcriptome | Allen Brain Atlas: BrainSpan project data |
| Cortex Cell Types Data Atlas | http://celltypes.brain-map.org/api/v2/well_known_file_download/694416044 |
| GTEx expression portal | https://gtexportal.org/home/gene/RCL1 |
|  |  |
| **Software and Algorithms** |  |
| Fiji/ImageJ | ImageJ, U. S. National Institutes of Health, Maryland |
| Zen Black/Blue | Zeiss |
| Adobe Illustrator CC 2017 | Adobe |
|  |  |
| **Antibodies** |  |
| NeuN | Millipore, MAB377 |
| *RCL1* | Sigma, SAB4500053 |
| *GFAP* | Abcam, ab7260 |
| *CTIP2* | Abcam, ab18465 |
| Alexa Fluor 488, 594, 650 goat anti-Mouse/Rabbit/Chicken | Invitrogen, 11001-10 |
|  |  |
| **Other** |  |
| SuperFrost Plus slides | Fisher Scientific,  Cat# 22-037-246 |
| ImmEdge Pen | Vector Labs,  Cat# H-4000 |
| Zeiss AXIO observer | Zeiss |
| Zeiss LSM700 confocal microscope | Zeiss |
| Leica Research Cryostat | Leica, Model # CM3050 S |
| 4’,6-Diamidino-2-Phenylindole, Dihydrochloride (DAPI) | Sigma-Aldrich, Cat# D9542 |

**SUPPLEMENTAL RESULTS**

**Figure S1**

Localization of RCL1 within the adult and fetal cortex.

**Figure S2**

*RCL1* is expressed within excitatory and inhibitory neurons in the adult neocortex, without signal in non-neuronal types


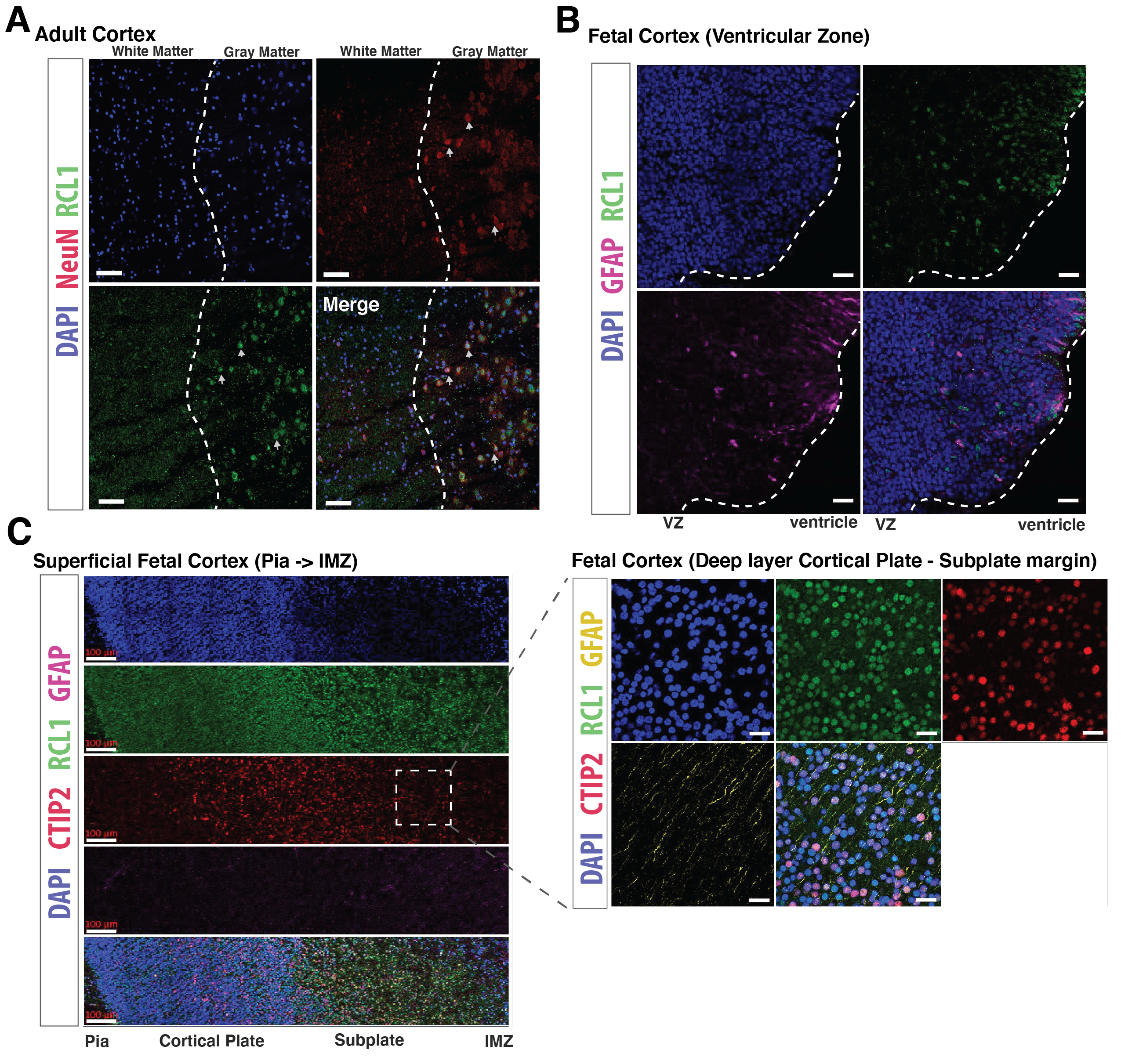


**Figure S1, Localization of RCL1 within the adult and fetal human cortex.**

**A.** Adult human brain tissue section with antibody staining for neuronal marker NeuN (RBFOX), and RCL1, demonstrate enrichment of RCL1 to neurons within gray matter, with little signal in white matter. Dotted line denotes separation of white and gray matter areas. Arrow indicate neurons co-expressing RCL1 with NeuN. Few cells within the white matter area express *RCL1*. Scale bar, 100μm. **B.** Ventricular zone area of a mid-gestation human fetal cortex tissue with anti-body labeling for cell type specific markers, GFAP+ progenitors and nuclei marker (DAPI) show RCL1 also present in the dividing cells of the ventricular zone. **C.** Cortical plate region of the mid-gestation fetal cortex depicting expression of RCL1 across several neuronal layers, including CTIP2+ deep layer neurons (Layers 5/6). Scale bar left, 100µm. *Right insert*, *RCL1* localized to neuronal somas in the cortical plate region, without significant co-labeling within GFAP+ fibers. Scale Bar 25 µm. IMZ, intermediate zone; VZ, ventricular zone; CP, Cortical plate.


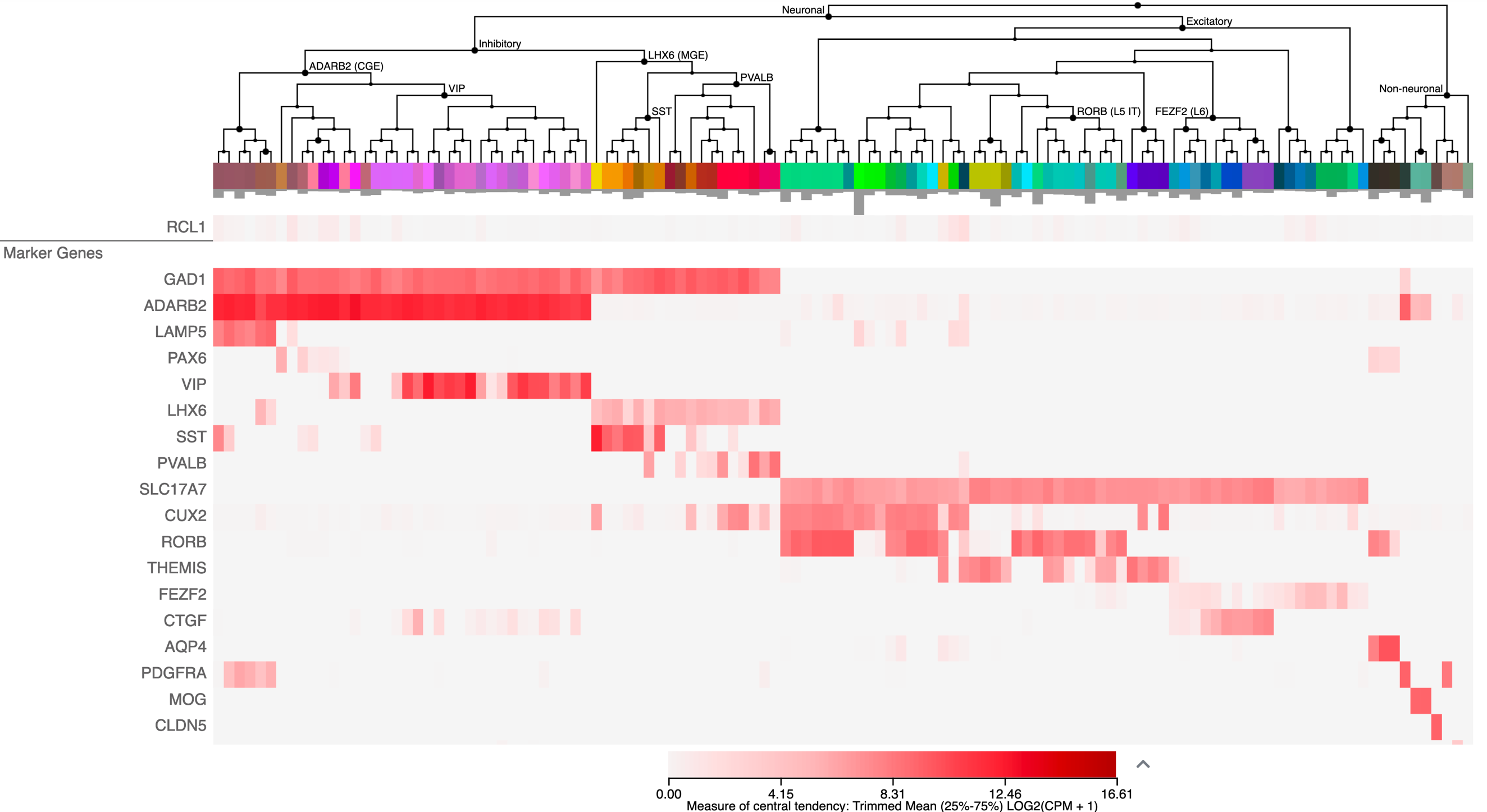


**Figure S2, *RCL1* is expressed within excitatory and inhibitory neuron subtypes in the postnatal human cortex, without signal in non-neuronal types**

Single cell RNA sequencing data of 49,494 single nuclei isolated from six areas of adult human cortex: middle temporal gyrus (MTG), anterior cingulate gyrus (CgGr), primary visual cortex (V1C), primary motor cortex (M1C), primary somatosensory cortex (S1C) and primary auditory cortex (A1C). Data downloaded from the Allen Brain Atlas Cell Types Dataset (see methods).
